# Over-the-Counter Sale of Antibiotics in India: A Qualitative Study of Providers’ Perspectives across Two States

**DOI:** 10.3390/antibiotics10091123

**Published:** 2021-09-17

**Authors:** Anita Kotwani, Jyoti Joshi, Anjana Sankhil Lamkang

**Affiliations:** 1 Department of Pharmacology, Vallabhbhai Patel Chest Institute (VPCI), University of Delhi, Delhi 110007, India; 2 Amity Institute of Public Health, Amity University, Noida 201301, India; jyoti@cddep.org; 3 Center for Disease Dynamics, Economics and Policy (CDDEP), New Delhi 110016, India; Sankhil@cddep.org

**Keywords:** antibiotics, antimicrobial resistance, dispenser, informal dispenser, informal provider, NAP-AMR, over-the-counter, pharmacist

## Abstract

India has one of the highest rates of antimicrobial resistance (AMR) worldwide. Despite being prescription drugs, antibiotics are commonly available over-the-counter (OTC) at retail pharmacies. We aimed to gain insight into the OTC sale of antibiotics at retail pharmacies and to elucidate its underlying drivers. We conducted face-to-face, in-depth interviews using convenience sampling with 22 pharmacists and 14 informal dispensers from 36 retail pharmacies across two Indian states (Haryana and Telangana). Thematic analysis revealed that antibiotics were often dispensed OTC for conditions e.g., fever, cough and cold, and acute diarrhea, which are typically viral and self-limiting. Both Access and Watch groups of antibiotics were dispensed for 1–2 days. Respondents had poor knowledge regarding AMR and shifted the blame for OTC practices for antibiotics onto the government, prescribers, informal providers, cross practice by alternative medicine practitioners, and consumer demand. Pharmacists suggested the main drivers for underlying OTC dispensing were commercial interests, poor access to public healthcare, economic and time constraints among consumers, lack of stringent regulations, and scanty inspections. Therefore, a comprehensive strategy which is well aligned with activities under the National Action Plan-AMR, including stewardship efforts targeting pharmacists and evidence-based targeted awareness campaigns for all stakeholders, is required to curb the inappropriate use of antibiotics.

## 1. Introduction

Antimicrobial resistance (AMR) is recognized as a global public health threat. With increasing AMR, especially antibacterial resistance in bacteria, common infections are becoming difficult to treat [1]. Rising AMR trends and the slow development of new antibiotics decrease the available options for the effective treatment of bacterial infections and also threaten the success of modern medical and surgical interventions. It is estimated that worldwide, 700,000 people die of drug-resistant infections every year, and this could increase to 10 million by 2050 if no proactive actions are taken to mitigate AMR [2]. Low- and middle-income countries (LMICs) will have a major share of AMR-associated economic and human losses as a high burden of infectious diseases co-exists with high rates of AMR [3,4,5].

The major driver for the rapid development and spread of antibiotic resistance is the total volume of antibiotic use, which exerts unnatural selective pressure on bacteria and potentiates the development of AMR [6]. A study indicated that between 2000 and 2015, antibiotic consumption (expressed in defined daily doses [DDD]) increased by 65% worldwide, largely driven by LMICs. In India, it increased from 3.2 to 6.5 billion DDDs (103%) [7]. Although the per-capita consumption of antibiotics in India is lower than that in several other countries [7], the proportion of broad-spectrum antibiotic consumption which are recommended for restricted use by the World Health Organization (WHO) is high [8,9].

This WHO classification consists of three categories of antibiotics—Access, Watch and Reserve (AWaRe). The goal is to reduce the use of the Watch group and Reserve group antibiotics and to increase the use of Access antibiotics [10]. Access group antibiotics show activity against a wide range of commonly encountered susceptible pathogens and have a lower potential of causing antibiotic resistance than the antibiotics in the other two groups. This includes antibiotics such as amoxicillin/ampicillin, benzathine penicillin and trimethoprim/sulfamethoxazole. The Watch group includes antibiotics with a higher resistance-causing potential than the Access group and includes antibiotics such as third-generation cephalosporins, fluoroquinolones and carbapenems. The Reserve group includes antibiotics that should be reserved only for the treatment of confirmed or suspected infections caused by multi-drug resistant organisms.

A recent study estimated that community antibacterial consumption comprised approximately 85–95% of the total antibacterial consumption in all the nations for which data were available [11]. Many studies suggest that a large majority of antibiotic use occurs in the community, where antibiotics are readily available and can be obtained without a prescription [12,13]. According to the WHO, over 50% of the antibiotic prescriptions worldwide are inappropriate with 2/3rds of antibiotics accessible at the pharmacies being used for self-medication [14] and the easy over-the-counter (OTC) access to antibiotics, which is common in LMIC’s [15]. There are a few recent reports showing that pharmacists dispensed antibiotics without prescriptions or that consumers visit pharmacies to buy antibiotics as OTC medicine, e.g., in Bangladesh [16], China [17], Mozambique [18], Thailand [19], and India [20]. Thus, self-medication and easy access to antibiotics without a prescription is one of the major concerns for the inappropriate and overuse of antibiotics in the community.

In India, the Drugs and Cosmetics Act, 1940, and the Drugs and Cosmetics Rules (DCR), 1945 designates all antibiotics as prescription drugs under the Schedule H category [21,22]. In 2014, an amendment was made to the Schedule H category to include second- and third-generation antibiotics into a new category called Schedule H1. For Schedule H1 drugs, pharmacists are required to maintain a separate register for the sale of these antibiotics and retain prescription copies [22]. This amendment was implemented to curb the widespread practice of antibiotic purchase from retail pharmacies without a valid prescription (old or outdated) or to prevent the pharmacists from dispensing antibiotics on their advice to the consumer/patient. However, several studies before and after 2014 indicate that consumers can still purchase antibiotics without a valid prescription as pharmacists still dispense antibiotics to their customers/clients/patients by attending to their symptoms [20,23,24,25].

Pharmacies in India are regulated under the provisions of the Drugs and Cosmetics Act, 1940 with Rules, 1945, which is a Central Act, passed by the Indian Parliament [21,22]. The sale, stock or exhibit for sale, supply, dispensing, and distribution of ‘drug’ is regulated under this Act/Rules, which provides that no person shall sell, stock or exhibit for sale, distribute or dispense any drug without holding a requisite license for the sale of drugs. The Drugs & Cosmetic Act/Rules provides for two categories of sale licenses (Retail and Wholesale) for supply chain management. Every retail sale licensee or pharmacy is required to have the services of a ‘registered pharmacist’ who may be the owner or employee of the pharmacy/licensee. ‘Registered pharmacists’ are governed under the provisions of the ‘Pharmacy Act’, 1948 [26] as far as their qualifications and registration as a ‘pharmacist’ is concerned. In India, a license for retail pharmacies is given to people with a diploma or degree in pharmacy. Indian drug laws categorize certain drugs as Scheduled G, H, H-1 and X drugs which cannot be dispensed without the prescription of a ‘Registered Medical Practitioner’ (Rule 65). There is no list of medicines defined like ‘OTC drugs’ under Indian law. However, antibiotics are clearly defined as prescription drugs under Schedule H and H1. Therefore, the dispensing of antibiotics by a pharmacist to a customer/patient without a valid prescription or on his/her advice is illegal and generally termed as an OTC sale of antibiotics. It is a common practice that retail pharmacies employ some salespeople who may not be trained in pharmacy (informal dispensers) to run the store and dispense medications [20]. All these pharmacies are private pharmacies and no medicine is available for free. Patients visit these pharmacies to purchase medication directly and avoid visiting a professionally trained doctor to save time and money [25].

AMR is a national priority and India has developed its National Action Plan on AMR (NAP-AMR) [27], well-aligned to the Global Action Plan on AMR. The NAP-AMR sets several Strategic Priorities, such as improving the awareness and understanding of AMR of all stakeholders through effective communication, education and training, optimizing the use of antimicrobial agents in the areas of health, animal husbandry and food, to list a few. The NAP-AMR outlines the need to explore the understanding, knowledge, and awareness regarding AMR and antimicrobial use amongst key stakeholders/target groups by conducting qualitative studies to understand their knowledge, attitudes, and practices. Few qualitative studies have been conducted in the country after the development of the NAP-AMR. While several studies have shown the widespread prevalence of OTC antibiotic dispensing in India, an understanding of the factors that drive this practice, especially from the perspective of dispensers at retail pharmacies, is lacking. Qualitative research provides a valuable opportunity to explore a deeper issue and gather the perspectives and lived experiences of the stakeholders involved holistically. Therefore, we conducted in-depth interviews of dispensers (pharmacists and informal dispensers) at retail pharmacies with an overarching aim of understanding their perspectives, reasoning, and the factors leading to the OTC sale of prescription drugs. The focus of the study was on the sale of antibiotics at retail pharmacies without a valid prescription of a doctor. This study was undertaken across two states of India, Haryana, located in the north, and Telangana, located in the southern part of the country. The objectives of the study were:

1. To assess the knowledge and awareness of antibiotics/AMR among dispensers at retail pharmacies;
2. To understand and uncover the drivers behind the practice of OTC antibiotic sales from the providers’ perspectives.

## 2. Methods

### 2.1. Study Design and Setting

A qualitative study was conducted to collect data from retail pharmacies in the 2 states of India, Haryana, which is in the northern part of India, and Telangana, located in the southern part of India. These 2 states have the 5th and 6th highest gross state domestic product in India, respectively [28], and are not amongst the top populous or largest states of India. From these states, districts with a high, medium and low Human Development Index (HDI) were selected. The HDI is a composite index of life expectancy, education and per capita income indicators, and it is used to rank cities into 3 tiers based on human development [29,30]. The 4 districts of Telangana: Hyderabad (capital city, high HDI), Warangal and Rangareddy (medium HDI), and Mahabubnagar (low HDI) and the six districts of Haryana: Chandigarh (capital city, high HDI), Panchkula and Faridabad (high HDI), Karnal and Sonipat (medium HDI), and Palwal (low HDI) were selected for the study. We chose to take an HDI index for sampling to cover populations belonging to different socio-economic strata of the states. This was conducted to capture variations (if any) in OTC treatment-seeking behavior based on their socio-economic condition. The study sites representing the 3 HDI-based tiers were selected based on logistic convenience.

### 2.2. Study Participants

Study participants were pharmacists/informal dispensers from retail pharmacies at the study sites. Study participants were approached at their respective retail shops and recruited using convenience sampling based on their willingness and availability to participate in the interview. One interview each was conducted in the selected retail pharmacy shop. In-depth interviews were conducted with 36 individuals (Telangana, 19; Haryana, 17; Table 1), of which 22 were qualified pharmacists (Telangana, 8 [T-P]; Haryana, 14 [H-P]); and 14 were informal dispensers with a non-pharmacy background (Telangana, 11 [T-ID]; Haryana, 3 [H-ID]). A minimum of 4 interviews were conducted in each type of HDI district, while in some other districts, more than 4 interviews were conducted unless we observed that the responses received were more or less similar and no new significant information emerged, indicating a saturation in response. In Telangana, 2 informal dispensers refused to participate in the interview because the owner was not present and they were unwilling to answer questions without the owner’s permission. In Haryana, 1 pharmacist was not comfortable answering the questions.

In Telangana, of the 11 informal dispensers, 4 had completed senior secondary schooling, 6 were graduates and 1 was currently pursuing a B. Pharmacy degree. Out of the 8 pharmacists, 3 had a diploma in Pharmacy (D. Pharma), another 3 had a Bachelor’s degree in Pharmacy (B. Pharm) and 2 had a Master’s degree (M. Pharm). The experience of informal dispensers varied from 6 months to 42 years, while that of pharmacists ranged from 6 to 20 years. In Haryana, all the pharmacists had diplomas; 5 of them had 15–20 years of work experience, while 3 had 3–5 years of work experience at a pharmacy. All the 3 informal dispensers had completed senior secondary school and had 8–10 years of work experience. All participants were male, with the exception of 3 female participants from Telangana (Table 1).

### 2.3. Development of Interview Guide for Semi-Structured In-Depth Interviews

A semi-structured interview guide (Supplementary File S1) was prepared by a team consisting of a social scientist (A.S.L.), community medicine specialist (J.J.), and a pharmacologist (A.K.) working in the field of antibiotic use and AMR. The guide was developed based on existing literature and the previous experience the A.K. has in such studies in India. The interview guide focused on the following domains: practice of OTC sale of antibiotics at the pharmacy, reasons for OTC sale of antibiotics, knowledge and understanding of antibiotics and antibiotic/antimicrobial resistance, and knowledge about existing regulations and guidelines regarding the OTC sale of antibiotics. These broad areas of knowledge were relevant to answering our research questions. Questions were then developed within these broad areas to tap into the participants’ experiences and perspectives. The interview guide was reviewed multiple times within the research team. Subsequently, it was pilot tested by the A.S.L., A.K. and 2 research fellows working on a project that focused on pharmacists in Delhi, and it was modified iteratively according to specific requirements. The interview guide was prepared in English and later translated into Hindi by a certified translation agency. Pilot-phase interviews also helped the interviewers realize that they must ensure that all the domains and questions have been covered by the end of the in-depth interview. The guide was kept flexible and included open-ended questions to encourage the exploration of domains that emerged from the interactive sessions. The refined interview guide with integrative flexibility was administered to the study participants to gain insights into the previously mentioned 4 domains.

### 2.4. Data Collection

Face-to-face, in-depth interviews were conducted using the semi-structured interview guide. Participants were asked open-ended questions, followed by additional probing questions. To avoid bias, the interviewers ensured to not overtly lead the participants while asking questions. A rapport was established with the participants prior to the commencement of the interviews. Data collection was carried out by 2 pairs of trained project research fellows between August 2019 and March 2020 in 2 languages (English and Hindi) according to the preference of the participants. The interviewers received training from the research team (A.K. and A.S.L.) before data collection. Hindi was the main language of the interviews/discussions in Haryana, while in Telangana, some of the interviews were conducted in Hindi and some were conducted in Telugu and later translated into Hindi or English with the help of a language translator. In concurrence with ethics considerations, informed verbal or written consent was obtained from the participants before the interviews, and interviews were also audio-recorded with the participants’ consent. The duration of each interview was 20–30 min. The participants’ confidentiality was maintained while conducting the interviews, analysis, and reporting.

### 2.5. Data Analysis

The interview recordings were transcribed verbatim and translated into English in MS Word for further analysis. All the transcripts were reviewed for accuracy by comparing them with the recordings. We used a reflexive thematic analysis approach to analyze the data. As described by Braun and Clark (2006), this analytical approach involves becoming familiarized with the data through an iterative process of reading the data-set transcripts, generating initial codes, arranging codes into larger categories, and drawing connections between codes and categories until a saturated thematic map can be generated [31]. The data were coded and analyzed in MS Word and MS Excel, and subsequent reviews were performed for thematic analysis. The coding process was both inductive and deductive, as some codes and themes emerged from the data, while some were derived from existing concepts and the interview guide framework. Each interview was independently coded by two research fellows, and comparisons were made to deliberate on any discrepancies in coding and arrive at a consensus. Once all the data were coded, the team of researchers (A.K., A.S.L., and research staff) reviewed them, and revisions were made wherever necessary to mitigate interpretative bias. All major themes and sub-themes were finalized with the consensus of the entire team.

The authors used the consolidated criteria for reporting the qualitative research (COREQ) checklist to develop the study methods, context of the study, findings, analysis, and interpretation [32].

## 3. Results

Using reflexive thematic analysis as proposed by Braun and Clarke (2006) [31], four themes with corresponding sub-themes were identified (Table 2). The respondents’ quotations are presented to illustrate the findings. Each respondent’s quotation was assigned the initials H for Haryana and T for Telangana, which were suffixed with P for pharmacists and ID for informal dispensers. These initials were followed by the respondent’s identity number.

### 3.1. Practice towards OTC Sale of Antibiotics

In-depth interviews with pharmacists and informal dispensers from Telangana and Haryana revealed patterns in the practice of the OTC sale of antibiotics, i.e., selling medicines without a valid prescription. This theme also sheds light on the factors that contribute to the OTC sale of antibiotics at retail pharmacies.

#### 3.1.1. Commonly Sold OTC Antibiotics for Minor Ailments

Most pharmacists and informal dispensers in Telangana were initially reluctant to admit that they dispensed antibiotics as OTC drugs. However, despite their denial, many of them were found dispensing antibiotics OTC during the interview (observational insights).

*“It is now compulsory to produce a prescription for almost all medicines. Even for PCM (paracetamol), nowadays, a warning (regarding consequences of overdose) comes on the backside of the strip of the medicine, and we should not be giving it without prescription.”* (T-P1)

Later, on further probing during the interview, they even mentioned names of commonly dispensed antibiotics for minor ailments. Strikingly, in Telangana, the informal dispensers were more cautious to admit that they sell non-prescription drugs as OTC drugs.

*“We don’t dispense. We only give it if they come with prescriptions. Generally, medicines for pain and fever are given as OTC.”* (T-ID9)

Some respondents, however, admitted to dispensing medicines including antibiotics for 1 or 2 days, dispensing 2–4 tablets/capsules of antibiotics to consume and advising them to visit a doctor if there are no improvements in their symptoms.

*“We do not give (antibiotics) for more than 1 or 2 days and customers also prefer to not take more.”* (T-ID2)

In contrast, pharmacists and informal dispensers in Haryana very clearly stated that they commonly dispense OTC antibiotics for common ailments, such as cold, cough, diarrhea, viral fever, sore throat, and ear infection. They further reported that sometimes patients directly approach them with symptomatic complaints for OTC medication. Thus, pharmacists often prescribe (symptomatic advice) and dispense antibiotics, generally for 2 days (instead of a complete course), and advise customers to seek professional help if they do not see improvements.

*“We give medicine for cold and cough and diarrhea. We give cefadroxil and amoxicillin-potassium clavulanate for sore throat and ear infection and observe the customer for 2 days, and then refer them to the doctor on the 3rd day.”* (H-P8)

*“We give ofloxacin, cefixime and sulfamethoxazole + trimethoprim in cases of sore throat.”* (H-ID3)

During the course of the discussion, the pharmacists and informal dispensers from both Haryana and Telangana mentioned the names of different medicines, including antibiotics, that they dispense for common ailments. Some common antibiotics dispensed were amoxicillin/clavulanic acid, azithromycin, ciprofloxacin, gentamicin, and cefixime. In addition, some fixed dose combination (FDC) antibiotics, such as norfloxacin + tinidazole + lactic acid bacillus and ofloxacin (200 mg) + ornidazole (500 mg) are also dispensed.

*“Azithromycin is mostly given for sore throat, while ciprofloxacin is given as ear drops. We also dispense gentamycin, ciprofloxacin, and other antibiotics depending on the nature of their health problem.”* (T-P4)

*“We give paracetamol along with a few antibiotics like chloramphenicol or ciprofloxacin, cefpodoxime, cefixime or ofloxacin for viral fever.”* (H-P9)

#### 3.1.2. Self-Medication with Old Prescriptions

Pharmacists and informal dispensers in Haryana reported that in most instances, consumers seek self-medication based on old prescriptions and knowledge/experience gained from a previous health episode. Pharmacists explained that they practice the demand-based sale of antibiotics. However, in Telangana, few pharmacists mentioned that consumers ask for antibiotics based on their prior knowledge.

*“The patients that come for self-medication straightaway ask for antibiotics and if we don’t dispense, they start arguing. Sometimes people come to buy medicines with an old prescription.”* (H-P13)

### 3.2. Factors Influencing OTC Sale of Antibiotics

This theme highlights factors that influence the OTC sale of antibiotics at retail pharmacies from the respondents’ perspectives. The details are discussed under each respective sub-theme.

#### 3.2.1. Lack of Easy Access to Public Healthcare Services

Respondents from Haryana stated the lack of easy access to public healthcare as one of the main driving forces for the spurt of OTC drug-seeking behavior in patients. The respondents mentioned that basic healthcare facilities in the country are inadequate and not easily accessible distance-wise. The number of public hospitals and doctors is not sufficient to cater to the population. In addition, the long queues in public healthcare facilities make it difficult for patients to visit these facilities for minor ailments. All these factors are deterrents against access to public healthcare services and also contribute towards patients’ OTC medication-seeking behavior. Retail pharmacy shops are thus felt compelled to dispense prescription drugs.

*“If all pharmacists refuse to give medicines without prescription, then there will be an outcry in India because the government doesn’t have enough facilities to provide for each patient. You know the scenario of civil hospitals (public); there is a big queue and they are always crowded. So, if a medical store person (retail pharmacies) stops giving medicines, everything will be at a standstill.”* (H-P10)

A few respondents from Telangana mentioned that instead of going to public healthcare facilities, a few patients directly come to them. However, this was not cited as one of the main reasons for OTC sales by the respondents from Telangana.

#### 3.2.2. Economic and Time Constraints

Respondents from Telangana emphasized the economic limitations of consumers, especially those from lower economic classes, who constitute the majority of the population in India, as one of the reasons for seeking OTC antibiotic dispensing. According to some respondents from both Telangana and Haryana, not everyone is ready to spend money on a doctor’s consultation for common or minor ailments. For such ailments, they seek OTC medicines from nearby retail pharmacies, and if their symptoms improve in 1–2 days, they avoid visiting doctors to save money and time.

*“It (OTC purchase of antibiotics/medicine) is a common practice since people don’t have enough money to go and consult a private sector doctor.”* (T-P1)

*“For minor illnesses, such as cold or cold, fever, headaches and body pain, they don’t want to spend money on the doctor’s consultation fee, so they just come for OTC medication without prescriptions.”* (T-ID11)

According to a few respondents from Haryana, the high cost of care at private healthcare facilities pushes people to seek medication from retail pharmacies.

*“People do not have the means to pay a doctor’s consultation fee.”* (H-P10)

*“People find this easier; doctor’s charge around INR 200, 300, and 500, and here the work is done in INR 20–30.”* (H-ID3)

In addition to the economic burden, the issue of time constraints was also highlighted. One of the pharmacists believed that consumers seek OTC medications to save time. They stressed upon the hectic schedule of working people and how OTC medication is commonly sought to get quick relief in the least time possible.

*“Everyone is working here and they have no time to go to a doctor for common ailments.”* (T-P1)

#### 3.2.3. Lack of Stringent Laws

Some of the pharmacists from Telangana mentioned that there are no stringent penalties to check for non-compliance of existing regulations, such as financial charges or license cancellations by the drug regulatory authority, which could otherwise deter non-compliance.

*“There are no financial penalties for OTC sale of antibiotics. But for other issues (regulations), there are penalties.”* (T-P5)

However, one of the respondents, an informal dispenser, disagreed and said that the violation of existing laws can result in penalties, such as the closing down of the pharmacy for a few days.

Pharmacists in Haryana also rued the lack of effective implementation of existing regulations, which results in non-compliance. They further stated that the penalty for violating regulations on antibiotic sales is not stringent enough, as only a word of warning and a temporary suspension of license is given by the Drug Inspector. However, there is no provision of fines, and only a show cause notice is issued to the pharmacists. Therefore, they opined that due to weak penalties and the absence of fines, the existing regulations are oftentimes violated at retail pharmacies.

*“There is no provision of fines; they just give a show cause notice to warn us and show why violating the regulations is inappropriate. But there is no monetary penalty for it, and as such, we only get a suspension for a few days, ranging from 2–3 days.”* (H-P12)

#### 3.2.4. Scanty Inspections

Respondents from both Haryana and Telangana further highlighted the scant visits from Drug Inspectors for inspection. They believed that the visits were made only when the inspectors received complaints, but even then, the inspectors perfunctorily and quickly checked a few minor issues and left. In Haryana, respondents also related the acute shortage of Drug Inspectors, as the number of retail pharmacies far outnumber them. Consequently, their workload increases and they cannot conduct regular inspections. Therefore, regulations are violated at almost every retail pharmacy.

*“All areas here come under Sonipat (a district in Haryana State) and there are about 500 chemist shops (retail pharmacies). After the inspection, they have to cover 5–7 companies and factories in that area. In addition, they have to appear for legal proceedings and other official work. So practically, they cannot perform inspections at retail pharmacies.”* (H-P10)

Moreover, informal dispensers in Telangana were unaware of any visits from Drug Inspectors and had no idea nor recalled the last visit from a Drug Inspector.

*“I don’t know, maybe once in a few months. I have not seen during my duty hours.”* (T-ID11)

#### 3.2.5. Safeguarding Commercial Interests

The pharmacists and informal dispensers from Haryana and Telangana shared similar thoughts with respect to commercial interests, i.e., profit generation. The constant consumer demand appeared to be an important factor for the sustained trend of the OTC sale of antibiotics. One of the pharmacists in Haryana reported that if a pharmacist refuses to sell OTC antibiotics, consumers can easily get it from another pharmacy. As such, they will lose customers, which will hamper their profits.

*“They will move to the next one. So, we lose the customer if we refuse to dispense antibiotics without a prescription.”* (H-P3)

Similar thoughts were echoed by pharmacists and informal dispensers from Telangana. They further explained the difficulties they encounter to keep their business afloat as their expenditure is high, compelling them to dispense OTC antibiotics.

*“Expenditure is high in small shops. So, they (referring to other pharmacists) dispense antibiotics for their business interests to meet the high expenses.”* (T-P5)

*“All chemists (pharmacies) do not follow each rule. Some pharmacists will give a strip of antibiotics costing INR 250 to increase their profit.”* (T-ID5)

### 3.3. Knowledge and Awareness of AMR and Antibiotic Regulations

This theme highlights the knowledge and awareness of pharmacists and informal dispensers with respect to antibiotics and AMR development. It further elaborates the understanding that pharmacists and informal dispensers have regarding existing regulations for the sale of antibiotics.

#### 3.3.1. Awareness and Practice towards Schedule H and H1 Drugs

Most pharmacists from both Haryana and Telangana were well-informed about the current regulations of the sale of antibiotics. They also had good knowledge of Schedule H and H1 drugs and the regulations around them, including the steps required to ensure compliance to the regulations.

*“For Schedule H1 drugs, we maintain the data of the drugs purchased and sold in a month. This record is checked by the Drug Inspector, and he also checks whether it (antibiotics) was given according to a prescription or not.”* (H-P14)

*“Schedule H1—yes we do keep records; we maintain all the details, name of the patient, their address and phone number, number of medicines given, etc.”* (T-P8)

However, despite their awareness, some pharmacists from Haryana still considered azithromycin as an OTC drug that could be sold without a prescription.

*“It (Azithromycin) is an antibiotic but it is a common medicine and it can be used over the counter.”* (H-P7)

On the contrary, knowledge about Schedule H1 was poor among informal dispensers, especially in Haryana. Two of the informal dispensers from Haryana were unaware of Schedule H & H1 guidelines, while the others were aware of the directives to be followed for the sale of antibiotics.

*“I don’t have any knowledge about Schedule H and H1.”* (H-ID3)

Most informal dispensers from Telangana were aware of the guidelines of Schedule H and H1 drugs.

*“Those medicines (Schedule H1 drugs) are on the list; they are given only with a doctor’s prescription. Information on how many tablets, for how many days, doctor’s name, patient’s name, mobile number, batch number, and the signature of the pharmacist are also required.”* (T-ID7)

#### 3.3.2. Knowledge about the Adverse Effects of Antibiotics

Some of the pharmacists from Telangana were aware of and knowledgeable about the adverse effects of antibiotics on the human body. They explicitly stated indigestion and allergies like redness, varying from person to person, as the adverse effects of antibiotics. They also stated that azithromycin, a common and popular antibiotic among consumers, causes fewer side effects than other antibiotics. However, several respondents from Haryana and informal dispensers from Telangana could not articulate any of the adverse effects of antibiotics.

*“If a patient takes more antibiotics, they will suffer from digestion problems. Antibiotics’ work is to kill bacteria; it does not matter whether it is good bacteria or bad bacteria. So, 100% of patients will suffer from digestion problems.”* (T-P2)

*“Yes, if we will take an antibiotic on our own, then it will have side effects (adverse effects), including an impact on kidneys.”* (H-ID2)

#### 3.3.3. Cognizance about Antimicrobial Resistance

Most pharmacists and informal dispensers from Haryana had a misconception regarding AMR. They believed that leaving an antibiotic regimen midway or taking suboptimal or extra doses of antibiotics would generate resistance against the drug in the human body, and not in microbes.

*“If it (antibiotic) is given in excess, then it will not be effective. If something is given repetitively, then the body will start resisting and get used to it.”* (H-P2)

Similar statements were made by pharmacists and informal dispensers from Telangana. They mentioned that the body develops resistance against the antibiotics. Further, they mentioned that the use of antibiotics for a longer period leads to the development of resistance in the body. One of the informal dispensers from Telangana mentioned that if antibiotics are consumed continuously, one’s immunity reduces. However, a few pharmacists correctly explained that it is the bacteria that become resistant.

*“Unnecessary use of antibiotics will make the body resistant to the drugs.”* (T-P7)

*“I don’t know much but the body naturally develops resistance against anything that is taken for a longer period, not just drugs. Like if people take sleeping pills for long, then gradually they don’t get sleep.”* (T-ID11)

### 3.4. Skirting/Dodging Responsibilities for OTC Sale of Antibiotics

This theme provides an insight into the perception of respondents about the practices of doctors, informal providers (IPs), alternative medicinal practitioners, and consumers in the community. Respondents believed that the irrational practices of prescribers impact the consumers’ behavior towards the OTC purchase of antibiotics.

#### 3.4.1. Doctors’ Prescription Practices Spurting OTC Sale of Antibiotics

According to the respondents from both Haryana and Telangana, doctors prescribe antibiotics inappropriately even in cases of cold and viral fever, where antibiotics are not required. Most of the respondents, especially pharmacists from Telangana, mentioned that the consumption and purchase of antibiotics have become high among consumers because doctors have prescribed them for an earlier health episode. Because doctors prescribe antibiotics irrationally for minor ailments, consumers assume that antibiotics can be used to treat any minor ailments. As a result, consumers usually seek OTC antibiotics for minor ailments based on an old prescription. This leads to the over-consumption and irrational use of antibiotics among consumers.

*“Suppose when you get a fever, you go to the doctor and they prescribe you at least one antibiotic when you can be treated with a simple paracetamol. The doctor will give you antibiotics like ciprofloxacin, ofloxacin or amoxicillin and clavulanate potassium.”* (H-P13)

Pervasive influence of Medical Representatives (MRs) of pharmaceutical companies over doctors’ prescriptions: Respondents also reported the influence an MR exerts over doctors’ prescription practices through financial incentives. They stated that doctors prescribe antibiotics and drugs from specific companies that send MRs to visit them instead of prescribing drugs that suit the patient’s medical or financial conditions. The doctors in turn receive financial or other incentives for prescriptions that help in increasing the sale of drugs/antibiotics for the MR, resulting in the over-prescription of antibiotics. This can further lead to a perpetual cycle wherein consumers who repeatedly receive unnecessary antibiotics may misconstrue antibiotics as OTC drugs to be used for minor ailments, spurting the demand for OTC antibiotic consumption.

*“Doctors will prescribe those medicines, including antibiotics, for which they receive visits from company representatives. Irrespective of whether it is needed for the ailment, several doctors will prescribe specific drugs to get financial incentives.”* (H-P12)

Distinction in prescriptions from public and private doctors: According to some pharmacists, there is a difference between the prescription patterns of public and private hospitals. Doctors from public hospitals prescribe fewer antibiotics, which is cost-effective and inexpensive for patients, whereas doctors from private hospitals prescribe a greater number of more costly antibiotics of a higher generation.

*“The prescriptions of private hospitals have more medicines as compared to those of civil hospitals. Civil hospital doctors prescribe three to four medicines and common antibiotics like ofloxacin, metronidazole, and amoxicillin and clavulanate, whereas private hospitals’ doctors prescribe higher antibiotics like meropenem. I have never seen so many medicines being prescribed in a civil hospital.”* (H-P13)

*“Doctors from public hospitals prescribe normal medicines like Cefixime and medicines that are inexpensive, but in private hospitals, costly medicines are prescribed at most instances.”* (H-ID1)

#### 3.4.2. Informal Providers’ Practice Causing OTC Sale of Antibiotics

Informal providers are unqualified medical practitioners or those without a license or registration to practice medicine. According to respondents, informal providers (IPs) dispense antibiotics inappropriately without a comprehensive knowledge and understanding of dose and duration. This is especially true in rural settings, where there is a lack of qualified medical doctors. Such irrational practices with inadequate knowledge influence consumers’ behavior, causing them to blindly follow prescribers and seek OTC antibiotics based on previous experience or an old prescription. Pharmacists see such irrational dispensing of antibiotics by IPs as equivalent to the OTC sale of antibiotics.

*“We are a developing country where the illiteracy rate is very high. You are collecting data here in Chandigarh, but if you go to villages, there are no doctors. There are either informal providers or quacks and they are prescribing medicines including antibiotics like doctors.”* (H-P3)

#### 3.4.3. Impact of the Cross-Practice of Alternative Medicine Practitioners

According to the respondents, the allowed cross-practice of allopathy by doctors with a Bachelor of Ayurveda, Medicine and Surgery (BAMS) degree spurts the irrational use of antibiotics in the community as they irrationally prescribe antibiotics of all classes. Hence, their irrational prescription of antibiotics is also equivalent to OTC antibiotic sales. In most instances, such practices influence consumer’s knowledge, attitude, and practice towards antibiotic use.

*“If somebody has an Ayurvedic degree and s/he is practicing allopathic medicine, then what is our government doing? The patient complains of stomach ache and says that they have taken medicines from a pharmacist but didn’t feel better, so the ayurvedic doctor gave them a stronger medicine (meaning next-generation antibiotic).”* (H-P3)

#### 3.4.4. Consumers’ Behavior

Most pharmacists from both states mentioned that consumers do not purchase a complete regimen of antibiotics. They stated that consumers tend to know basic medicines from experience and therefore directly ask for OTC antibiotics. If the same symptoms from a previous health episode re-appear, they come to buy the same antibiotic. In the process, they may take an incomplete regimen again. Consumers tend to stop taking drugs soon after getting better, disregarding the regimen duration.

*“You know the complete course (regimen) of antibiotics will be 3–5 days, but generally customers do not purchase the complete course of antibiotics. They purchase medicines only for 2 days.”* (T-P3)

Respondents explained that when a doctor prescribes antibiotics for 5 days, patients rarely complete the regimen. The inappropriate behavior of antibiotic consumption is quite prevalent, irrespective of the educational status of the consumers. One pharmacist explained that an uneducated customer sometimes takes an extra dose of antibiotics while an educated customer tends to purchase antibiotics insufficient for the complete regimen.

*“People take an incomplete course even if the doctor has prescribed it for 5 days. They take it only for 2 days and then leave it. Later they come to return the medicines saying they have recovered in 2 days only. Then, we advise them to take the complete course for the next 3 days even if they have recovered.”* (H-P10)

## 4. Discussion

This study provides findings on the practice of OTC antibiotic sales and its underlying causes using a comprehensive qualitative survey performed among dispensers across two Indian states. In-depth interviews with 36 respondents (22 pharmacists and 14 informal dispensers) across Haryana and Telangana showed that antibiotic dispensing without a prescription by pharmacists and dispensers to clients, consumers, and patients is common. The retail pharmacies must have at least one qualified pharmacist to obtain a license. However, most retail pharmacies have unqualified personnel who run the business and dispense medicines, including “OTC antibiotics.” This is a common practice, as reported earlier in a study from New Delhi, India [20,25].

### 4.1. Pattern of Antibiotics Dispensed

Our study found OTC antibiotic dispensing to be a common practice among pharmacists and informal dispensers in both survey states, Haryana and Telangana. These antibiotics were usually dispensed for viral and self-limiting conditions, including fever, cold, cough, and sore throat. These practices of antibiotic dispensing clearly indicate that retail pharmacies not only flout the rules for dispensing drugs (both Schedule H and H1) without a prescription, but also give antibiotics in cases where they are not required. Earlier studies have indicated that consumers can easily obtain antibiotics without a prescription in LMICs [13,25,33].

Pharmacists stated that the common antibiotics they dispense include cefradroxil (first-generation cephalosporin; Access group), amoxicillin + potassium clavulanate (Beta lactam + Beta Lactam inhibitor; Access group), ofloxacin (Fluoroquinolone; Watch), cefixime (third-generation cephalosporin; Watch group) and sulphamethoxazole + trimethoprim (trimethoprim and sulphonamide combination; Access group). Other common antibiotics dispensed included azithromycin (Macrolide; Watch group), ciprofloxacin (Fluoroquinolone; Watch group) and gentamicin (Aminoglycoside; Access group). Respondents also mentioned dispensing FDC antibiotics, such as norfloxacin + tinidazole + lactic acid bacillus and ofloxacin [200 mg] + ornidazole [500 mg]. These Fixed-Dose Combinations (FDCs) are commonly dispensed for acute diarrhea [20] and contain a fluoroquinolone from the Watch group of antibiotics and these FDCs are not rational combinations. Moreover, respondents also mentioned that they dispense antibiotics on their own to provide patients with symptomatic relief for 1 or 2 days. This practice of antibiotic use for a brief period also constitutes the inappropriate use of antibiotics and leads to the rapid development of AMR. Respondents dispensed not only Access group antibiotics but also Watch group antibiotics, which could also lead to the rapid development of AMR, as mentioned by the WHO [10]. Previous studies have indicated that the consumption of Watch group antibiotics is increasing in India as compared to Access group antibiotics [8,9].

Respondents in our study mentioned dispensing antibiotics and FDCs for common acute diarrhea. This is similar to findings from studies conducted earlier in New Delhi [20] and Goa [33] wherein antibiotic dispensing for acute conditions was observed. The use of FDCs is irrational and they are not included in any list of essential medicines, including the National List of Essential Medicines, India [34].

### 4.2. Access to the Healthcare System and Universal Health Coverage (UHC)

One of the important factors respondents mentioned for why consumers/patients visit retail pharmacies for common ailments was the poor access to public health facilities and non-affordable fees for private sector doctors. Previous studies have highlighted that public health facilities are not accessible to a majority of the population, and most Indians rely on the expensive private sector for their healthcare needs [35]. It takes almost a day for patients to receive treatment from a public facility, making it difficult for people from economically-disadvantaged classes, such as daily-wage laborers, to lose one day of wages in order to receive free treatment. India has one of the highest rates of out-of-pocket (OOP) expenditure in the world [36]. Recent data from the government indicates that primary care comprises 41% of the private OOP household expenditure on health in India [37], and an overall 68% of outpatient care in rural areas and 74% in urban areas is sought in the private sector [38]. Therefore, it was not surprising that our study respondents mentioned the lack of access to adequate public healthcare facilities as one of the key drivers for OTC antibiotic dispensing. Moreover, it is difficult for most of the Indian population to afford the high cost of treatment in the private sector. Economic and time constraints are important factors that cause patients to visit retail pharmacies for treatment. In our study, we found that one key driver for seeking OTC antibiotics was the fear of the high cost of care, especially among individuals from lower socio-economic classes. Another factor was the lack of time for a detailed medical consultation among the consumers. Similar findings have also been reported in Nepal [39], where OTC antibiotic use is driven by the intention of dispensers to prevent the cost of consultation and travel to the hospital, and patients also save money by bypassing the formal system of care.

UHC, which enables sustainable, equitable and affordable access to healthcare and ensures access to medicines may decrease the number of patients directly visiting a retail pharmacy to purchase antibiotics. It is essential to take a systems-oriented approach to medicine access rather than viewing this access as only a series of interactions between patients and public health services [40]. The Ayushman Bharat scheme of the government of India [41] is a step towards providing UHC. The Ayushman Bharat scheme has two important components; provision of comprehensive primary healthcare (CPHC) that covers maternal and child health services and non-communicable diseases, including free essential drugs and diagnostic services and the Pradhan Mantri Jan Arogya Yojna or PM-JAY aims to provide health insurance coverage of 500,000 INR (6822 USD) per family per year for secondary and tertiary care hospitalization of poor and vulnerable (bottom 40%) Indian families. However, the scheme has been criticized for being unbalanced and skewed towards costly inpatient care instead of protecting people from outpatient costs, including the purchase of drugs such as antibiotics, which make up 70% of India’s healthcare-related OOP expenditure [42].

### 4.3. Non-Strict and Poor Implementation of Regulations

In our study, respondents highlighted that there are no financial charges as a penalty for the OTC sale of antibiotics. There are few stringent penalties for dispensing antibiotics without a prescription. Usually, a show cause notice is sent for flouting this rule, and in rare cases, the shop is shut down for a day or two. Moreover, as the inspections are not regular, there are few deterrents against dispensing antibiotics as OTC drugs. The lack of fines and infrequent inspections by regulators emerged as critical factors driving OTC antibiotic dispensing. Our earlier study revealed that poor awareness among consumers increases the OTC purchase of antibiotics. Consumers have little awareness that antibiotics are prescription drugs [25] and therefore buy them without consulting a doctor. For pharmacists, such self-medication and antibiotic dispensing are an important part of business, and they do not wish to lose their clients [23].

There is a paucity of research on the impact of law enforcement activities in reducing OTC access to antibiotics in LMICs. A systematic review [43] on the impact of interventions undertaken in LMICs reported the varied effect of a range of law enforcement activities in decreasing OTC antibiotic dispensing. These measures included government inspections (regulatory interventions), involvement of pharmacists in designing interventions and retention of prescriptions (managerial interventions), and media campaigns and education for pharmacists (educational interventions). Systematic review findings revealed a large variation in the impact of different law enforcement activities in different countries. Data from several countries have measured either a change in antibiotic use in general or in a specific class of antibiotics. The outcome was documented to be either predominantly significantly positive or having no effect, but few reports also show conflicting evidence. The impact of these interventions was difficult to quantify as multiple methods with different outcome measures have been used in different countries. These divergences in settings and methods make it hard to compare outcomes and determine the effectiveness of specific law enforcement activities [43].

### 4.4. Poor Knowledge and Awareness about Antibiotic Regulations and AMR

Knowledge is a key driver of professional practice, and pharmacists are the gateway to not only medicine use but to the larger healthcare system. When access to healthcare is limited (as in LMICs like India), pharmacists not only dispense medicines but also play a key role as stewards for appropriate antibiotic use in the community [44,45,46]. In our study, we found that most pharmacists were largely aware of the regulations of Schedule H and H1 in India, under which certain drugs are defined as prescription-only drugs. However, not all pharmacists were aware of the adverse effects of antibiotics and could describe only few such effects in detail.

Most informal dispensers were poorly informed on the topic and expressed ignorance about current regulations. We interviewed 14 (of 36) informal dispensers working in pharmacies at the time of the interview and found that most of them from Haryana did not have adequate knowledge regarding antibiotics and Schedule H or H1. Though the practice of employing additional staff (untrained in pharmacy or medicine and usually in dispensing and managerial roles) is common in pharmacies in India and many other Asian countries, very few studies have assessed their level of knowledge [45]. These informal dispensers are not trained in pharmacy, making it likely that their knowledge about rules, antibiotics, and AMR is not sufficient. As per the DCR [22], a registered participant must always be present in the pharmacies. However, it is common to see untrained personnel (informal dispensers) dispensing medicines in pharmacies across countries in Asia, including India [47,48,49].

Several studies in LMICs, such as Tanzania [44], Sri Lanka [45] and Pakistan [46] and even a high-income country like Saudi Arabia [50], have demonstrated poor knowledge of antibiotic use and ignorance over antibiotic resistance and regulations on antibiotic dispensing among pharmacists. These clearly indicate the important need to educate pharmacists and informal dispensers regarding antibiotic use (mode of action, rise of antibiotic resistance, and value of regulations) considering their key role in ambulatory care and direct contact with patients. Studies have demonstrated that sensitized and professionally educated pharmacists dispense fewer antibiotics for suspected viral infections, such as upper respiratory tract infections [45].

#### Knowledge of AMR

Surprisingly, the correct understanding of AMR and how it develops was absent in most pharmacists and informal dispensers. This is particularly concerning for pharmacists who have been trained in pharmaceuticals and medicines and are expected to be a medium for spreading awareness on appropriate medicine use. Messaging about AMR needs to be reinforced to both pharmacists and informal dispensers, as most of them interpreted it to be resistance in the human body and immune response rather than a change in the susceptibility of disease-causing pathogens, like bacteria, that causes them to survive antibiotic treatment. Our findings are similar to those of previous studies conducted in India in New Delhi [23] and Bangalore [51].

### 4.5. Shirking Responsibility and the Blame Game

The behavior and practices of peers and other healthcare providers in the community emerged as some of the key factors that drove OTC antibiotic dispensing according to the respondents. Inappropriate prescriptions from doctors—including IPs as well as trained medical practitioners, especially in the private sector owing to the influence of pharmaceutical company representatives—was cited as a common practice that prompted the demand of antibiotics among consumers, even for minor ailments.

IPs are a range of providers without a formal degree in medicine who are not registered as healthcare practitioners. They include a range of actors, such as unqualified doctors, spiritual healers, and traditional birth attendants and are the most common health providers in rural India [52]. IPs commonly prescribe allopathic medicines, including antibiotics, in addition to other traditional medicines [53]. The respondents of our study reported this phenomenon, which has also been validated by other studies in India, in which IPs from rural areas of Madhya Pradesh [54] and West Bengal [55] were found to prescribe a high number of antibiotics. The Madhya Pradesh study [54] found that IPs prescribe a higher proportion of antibiotics (74%) than that of other non-antibiotic medications for common illnesses. The respondents of our study opined that the prescription of antibiotics by IPs is equivalent to OTC antibiotic dispensing as IPs do not have any legal rights to prescribe or dispense antibiotics.

With a shortfall of 1.8 million health workers (doctors, nurses, and midwives), India lags behind the WHO target of having 44.5 health workers per 10,000 population by 2030 [56]. While the number of doctors, nurses, and midwives is half of the prescribed WHO norm (24.5 per 10,000 population), healthcare in India is serviced largely by unqualified practitioners, and a large number of Indians, especially rural residents and poor urban residents, receive care from unqualified or under-qualified providers [57,58].

The lack of UHC for most Indians and the burden of OOP expenditures mean that most Indians who live in rural areas rely on IPs for healthcare services. Studies have shown that broad-spectrum antibiotics are the most commonly prescribed drugs by IPs, and this further contributes to the inappropriate use of antibiotics in the community and rising AMR trends [52].

### 4.6. Strengths, Challenges and Mitigation

To our knowledge, the present study is the first to obtain an in-depth insight into OTC antibiotic dispensing in India from the perspective of both pharmacists and informal dispensers across two Indian states located in the north and south. Furthermore, the study was comprehensive as it looks at districts with different HDIs to capture variations (if any) in OTC treatment-seeking behavior across populations belonging to different socio-economic strata.

Nevertheless, the research team encountered a language barrier in Warangal and although a local pharmacist was hired as an interpreter, the research team was unable to communicate directly to those two participants. As such, the interview got disrupted with back-and-forth interpretations. Some of the participants were initially giving perfunctory and diplomatic responses on the OTC dispensing of antibiotic practices because of its legal implications. However, after rapport building and further probing, the researchers were able to generate genuine responses that reflected ground reality.

## 5. Conclusions

In our study conducted across two Indian states, the inappropriate OTC dispensing of antibiotics by pharmacists and informal dispensers in retail pharmacies was found to be common. We observed a low awareness about AMR and the adverse effects of antibiotics among dispensers. According to the dispensers, the primary systemic factors causing poor practices of OTC antibiotic dispensing were poor access to healthcare services and inappropriate prescriptions by qualified health providers and by informal and unqualified health providers with no knowledge of antibiotics. Moreover, the respondents believed that the consumer demand and financial interests of business owners further added to this phenomenon. Given the rising trend of AMR in India and the relationship between OTC antibiotic dispensing and AMR, there is an urgent need for stewardship efforts targeting dispensers at pharmacies, prescribers, as well as consumers in order to reduce OTC antibiotic dispensing in the country. A systems-oriented approach needs to be taken to tackle OTC sales of antibiotics as the situation cannot be resolved just by interventions targeted at retail pharmacies alone. Targeted awareness campaigns aimed at improving the understanding of AMR and the appropriate use of antibiotics and monitoring behavioral changes among all stakeholders, including prescribers, consumers and pharmacists, are expected to be helpful. More importantly, the strict enforcement of regulations for prescription-based antibiotic dispensing and innovative regulatory practices could be key for reducing OTC antibiotic dispensing in India and help the country meet its NAP-AMR goals.

## Figures and Tables

**Table 1:** Demographic details of respondents at retail pharmacies in Haryana and Telangana.

Characteristics	Haryana		Telangana	
Age Group	Pharmacists	Informal Dispensers	Pharmacists	Informal Dispensers
18–24 years	1	-	-	2
25–35 years	2	1	3	3
35–50 years	9	2	3	5
>50 years	2	-	2	1
Gender				
M	14	3	6	10
F	0	0	2	1
Education				
Senior Secondary School	0	3	0	4
Diploma in Pharmacy	14	0	3	
Pharmacy Graduate	0	0	3	6 (non-pharmacy) + 1 pursuing a B. Pharm degree
Pharmacy Post-graduate	0	0	2	0
Experience				
<5 years	2	-	-	2
5–10 years	3	3	2	6
>10 years	9	-	6	3
Total	14	3	8	11

**Table 2:** Themes and sub-themes derived through systematic thematic analysis of interviews.

Theme	Sub-Themes
Practice towards over-the-counter (OTC) sale of antibiotics	Commonly sold OTC antibiotics for minor ailments; Self-medication with old prescriptions.
Factors influencing OTC sale of antibiotics	Lack of easy access to public healthcare facilities; Economic and time constraints; Lack of stringent laws; Scanty inspections; Safeguarding commercial interests.
Knowledge and awareness of antimicrobial resistance and antibiotic regulations	Awareness and practice towards schedule H and H1 drugs; Knowledge about the adverse effects of antibiotics; Cognizance about antimicrobial resistance.
Skirting responsibilities for OTC sale of antibiotics	Doctors’ prescription practice spurting OTC sale of antibiotics; ⮚ Pervasive influence of Medical Representative of pharmaceutical companies over doctors’ prescriptions; ⮚ Distinction in prescriptions from public and private doctors; Informal providers’ practice causing OTC sale of antibiotics; Impact of the cross-practice of alternative medicine practitioners; Consumers’ behavior.

## Data Availability

Aggregate de-identified respondents’ data will be available after the publication of this study. Requests should be sent to the corresponding author, who will discuss the request with the team and decide whether the data should be shared based on the feasibility, novelty, and scientific rigor of the proposal. All applicants will be required to sign a data access agreement.

## Supplementary Materials

The following are available online at https://www.mdpi.com/article/10.3390/antibiotics10091123/s1, Supplementary File S1: Interview Guide.
