# Supplementary material for: Over-the-Counter Sale of Antibiotics in India: A Qualitative Study of Providers’ Perspectives across Two States
Source: Antibiotics (Basel). 2021 Sep 17;10(9):1123. doi: 10.3390/antibiotics10091123 (PMC8472180; doi:10.3390/antibiotics10091123)
Supplement: Supplementary file 1 [file antibiotics-10-01123-s001.zip › antibiotics-1339909-supplementary.pdf]

Semi-structured interview guide (Theme based) for pharmacists (Over-the-counter sale of antibiotics)

Instructions for the Interviewer (IR)

1) *The Participant Information Sheet (PIS) should be given to the respondent and the consent form read out and signed/verbally agreed with a witness. Seek permission to record the interview after the PIS and signing/agreeing of the consent form.*
2) *Seek interviewee's (IE's) opinion as much as possible, do not prompt responses/avoid asking leading questions.*
3) *This interview guide is only for reference; the interviewer can modify sequence of questions or ask additional questions depending upon the situation.*
4) *It is advisable that not to use the abbreviations like Over the Counter (OTC), and Antimicrobial Resistance (AMR) during the course of Interview.*
5) *Please note carefully the names of medicine -either brands or generic compounds as told by the interviewee/respondent. Ask for the exact spelling if not clear.*

NOTE: *Approach the respondent (Interviewee- IE, Interviewer - IR) in a non-intimidating way. You may observe some of the interactions of the respondent in dispensing at the pharmacy for a while before approaching directly. Initiate casual conversation with courtesy by introducing yourself and asking the same to make the respondent feel at ease. Once you feel that the respondent is comfortable, explain the purpose of your visit and ask if s/he is willing to participate in the study using participant information sheet (PIS) and consent form (CF). Once you are done with the PIS and CF, start the interview by collecting the following basic information:*

A. Introductory questions

1. Introduction and rapport building: Name, educational qualification, work experience.

2. Information of the other staff working in the pharmacy.

Probe: Qualification/ Designation and training in the field

3. How long has this pharmacy been established and what range of medicines are available in the pharmacy?

B. Theme: Practices and perceptions of over-the-counter (OTC) sale of medicines in the pharmacy

4. What are the common ailments for which medicines are directly sought by consumers at your pharmacy without prescription? (Do not specify symptoms or illnesses)
5. What are some of the most common medicines being directly purchased in your pharmacy without prescription? Are these injectable/oral/ topical applications?

a) *Can you name some medicines that have been highly in demand over the last six months?*
b) *If s/he mentions antibiotics, emphasize on the antibiotics mentioned and probe further about them (what are these antibiotics used/given for or what are the illnesses/symptoms for which these antibiotics are used?).*

6. Usually, what are the trends with respect to sale of medicines in different seasons and changes in the weather?

Probe:

a) *Are there any fluctuations in seasonal/weather/festival related sale of medicines? Can you please elaborate this?*
b) *Talk about medicines for minor illness like fever, diarrhea and cold or cough etc. and prompt the names of medicines usually used for treating these illnesses (not necessarily stressing on antibiotics)*
c) *Any seasonal variations for antibiotics? If pharmacist has listed some antibiotics, then ask about their dispensing and prescription practices.*
d) *Check if the antibiotics (which s/he has named) are sought by the patients or suggested by the pharmacist? Generally, which medicines are given/suggested by pharmacist?*
e) *Ask the names of some of the medicines generally given by pharmacist for patients with symptom of cough, cold, fever etc. for 2-3 days? (if antibiotics are named, probe why they think these antibiotics (use the names of antibiotic mentioned) would be helpful for these illnesses).*

7. How often do you receive consumers who ask for medicines over the counter? Please recall the names of the medicines usually requested (Check if any of the mentioned names are antibiotics and probe for which symptoms are these requested?)

Probe:

a) *Make sure to ask how many tablets or capsules do they buy compared to the course. Probe for medical advice on dose and duration and if advice is being sought from them on these.*
b) *What type of consumers (age group, gender, education, or other socio-economic and other demographic characteristics) come for buying antibiotics over the counter?*

C. Theme: Understanding about antibiotics and resistance to antibiotics, about newer antibiotics being developed and AMR Knowledge

8. Can you explain what are antibiotics?

Probe: What are antibiotics used for and how antibiotics act?

9. Are new antibiotics coming into the market? If yes, can you tell us names of some of the new antibiotics? Which companies are making these new antibiotics?

Probe: Why do you think there is a need for new antibiotics?

10. Have you heard of the term Antimicrobial Resistance (AMR)? Or antibiotic resistance? How does it develop?

Probe: If yes, can you tell us what is AMR?

11. Do you think antibiotic resistance is important?

Probe:
a) *If yes, what are the main causes of increasing AMR?*
b) *If they say, there is not much resistance, then ask if they have heard/read in the newspaper about increasing resistance. What are his/her views on it?*

12. Do you see any role of over-the-counter sales of antibiotics leading to antibiotic resistance?

D. Theme: Knowledge and perception about existing regulations and guidelines on OTC use of antibiotics

13. Are there any rules/guidelines/protocols to manage sale and purchase of medicines at your pharmacy? Where did you learn them (during the discussion, focus upon antibiotics)?

Probe:
a) *Are there any government guidelines for selling antibiotics?*
b) *Depending upon the answer, probe about Schedule H and H1.*

14. How do you get updates or changes in these rules or regulations?

15. Are there any regular programs for pharmacists where scientific updates about current rules and regulations for medicines are provided? Where are these conducted? Who conducts such programs or meetings?

Probe:
a) *Have you attended any Continuous Pharmacy Education (CPE) updates in the recent past? When was it?*
b) *Is there any compulsion towards attending CPEs?*
c) *What all aspects were discussed in the CPE? Has AMR, regulations and guidelines for antibiotics usage and dispensation been discussed in these meetings?*

E. Theme: Perception about gaps between existing regulations and poor compliance

16. How often do Drug Regulators (inspectors) regulate or conduct inspections/checks of pharmacies?

Probe: How regularly have drug inspectors visit your pharmacy?

17. What do Drug Inspectors usually check when they visit your pharmacy?

Probe:
a) *When was the last visit you encountered from a drug inspector? Is it frequent/infrequent?*
b) *Usually what are the reasons for punishment or fines in the visit?*
c) *How large are the fines/actions for any unlawful offense at pharmacies?*
d) *Why do you think that fines are given? What are the rules thatare not followed in pharmacies? (Probe especially regarding prescription-only sale of antibiotics)*
